# CITEViz: interactively classify cell populations in CITE-Seq via a flow cytometry-like gating workflow using R-Shiny

**DOI:** 10.1186/s12859-024-05762-1

**Published:** 2024-04-02

**Authors:** Garth L. Kong, Thai T. Nguyen, Wesley K. Rosales, Anjali D. Panikar, John H. W. Cheney, Theresa A. Lusardi, William M. Yashar, Brittany M. Curtiss, Sarah A. Carratt, Theodore P. Braun, Julia E. Maxson

**Affiliations:** 1 Division of Oncologic Sciences, Knight Cancer Institute, Oregon Health and Science University, 3181 SW Sam Jackson Pk. Rd., KR-HEM, Portland, OR 97239 USA; 2 Earle A. Chiles Research Institute, Providence, Portland, OR 97213 USA; 3 Knight Campus Graduate Internship Program - Bioinformatics, University of Oregon, Eugene, OR 97403 USA; 4 Cancer Early Detection Advanced Research, Oregon Health and Science University, Portland, OR 97238 USA; 5 Department of Biomedical Engineering, Oregon Health and Science University, Portland, USA; 6 Division of Hematology and Medical Oncology, Oregon Health and Science University, Portland, USA

**Keywords:** CITE-Seq, Single-cell RNA-Seq, scRNA-Seq, R-Shiny, Single-cell, Multi-omic, Flow cytometry, Cluster classification

## Abstract

**Background:**

The rapid advancement of new genomic sequencing technology has enabled the development of multi-omic single-cell sequencing assays. These assays profile multiple modalities in the same cell and can often yield new insights not revealed with a single modality. For example, Cellular Indexing of Transcriptomes and Epitopes by Sequencing (CITE-Seq) simultaneously profiles the RNA transcriptome and the surface protein expression. The surface protein markers in CITE-Seq can be used to identify cell populations similar to the iterative filtration process in flow cytometry, also called “gating”, and is an essential step for downstream analyses and data interpretation. While several packages allow users to interactively gate cells, they often do not process multi-omic sequencing datasets and may require writing redundant code to specify gate boundaries. To streamline the gating process, we developed CITEViz which allows users to interactively gate cells in Seurat-processed CITE-Seq data. CITEViz can also visualize basic quality control (QC) metrics allowing for a rapid and holistic evaluation of CITE-Seq data.

**Results:**

We applied CITEViz to a peripheral blood mononuclear cell CITE-Seq dataset and gated for several major blood cell populations (CD14 monocytes, CD4 T cells, CD8 T cells, NK cells, B cells, and platelets) using canonical surface protein markers. The visualization features of CITEViz were used to investigate cellular heterogeneity in CD14 and CD16-expressing monocytes and to detect differential numbers of detected antibodies per patient donor. These results highlight the utility of CITEViz to enable the robust classification of single cell populations.

**Conclusions:**

CITEViz is an R-Shiny app that standardizes the gating workflow in CITE-Seq data for efficient classification of cell populations. Its secondary function is to generate basic feature plots and QC figures specific to multi-omic data. The user interface and internal workflow of CITEViz uniquely work together to produce an organized workflow and sensible data structures for easy data retrieval. This package leverages the strengths of biologists and computational scientists to assess and analyze multi-omic single-cell datasets. In conclusion, CITEViz streamlines the flow cytometry gating workflow in CITE-Seq data to help facilitate novel hypothesis generation.

**Supplementary Information:**

The online version contains supplementary material available at 10.1186/s12859-024-05762-1.

## Background

The development of high-throughput single-cell RNA-Sequencing (scRNA-Seq) methods has revealed previously unappreciated levels of cellular heterogeneity [1]. Since the development of Drop-Seq in 2015, a number of scRNA-Seq assays have been developed to profile multiple macromolecules in the same cell. For example, CITE-Seq is a multi-omic variant of scRNA-Seq that captures the cell surface proteome using antibody-derived tags (ADT) [2]. Multi-omic assays like CITE-Seq introduce new dimensionality to the data, but often require nuanced analyses to extract meaningful results. For example, a common single cell analysis consists of unsupervised clustering of cells followed by the classification of cell populations. However, there can be low correlation of differential gene signatures and cell cluster identity, and the process is often time-consuming and irreproducible. By using the ADT information in CITE-Seq data, we aim to approximate a flow cytometry workflow to better classify cell clusters based on known surface identity markers.

The gating workflow in flow cytometry is the gold standard to classify cell populations using cellular surface protein markers. During a typical flow cytometry experiment, fluorescently-labeled antibodies stain cell surface proteins, and individual cells are identified based on fluorescent signal intensity [3]. Cells are then plotted in two dimensions based on surface marker abundance (e.g. CD38 and CD34), and boundaries are drawn (called gates) around cell populations of interest. Selected cells are further re-plotted in a new set of surface markers and filtered again until a population of interest is identified and quantified [4]. The gating workflow is essential to investigate biological perturbations (e.g. drug treatment in cancer cells) in which the proportional changes in cell populations are tracked. The same principles of gating can be applied to CITE-Seq [5], but to the best of our knowledge, the bioinformatics field currently lacks a robust program that facilitates this interactive process in Seurat-processed CITE-Seq data.

To efficiently gate cell populations in CITE-Seq data, we developed CITEViz. By using the R-Shiny platform, CITEViz allows users to interactively subset cell populations of interest using surface proteins and see those cells highlighted in latent space (e.g. PCA, tSNE, UMAP). Conversely, cell clusters can be selected in latent space and quickly located in a 2D feature scatter plot (called a back-gate). A secondary function of CITEViz is to provide basic multi-omic single/co-expression feature plots and quality control figures to allow biologists to quickly and holistically assess CITE-Seq data. In conclusion, CITEViz (1) streamlines the gating workflow in CITE-Seq data to identify cell populations and (2) facilitates basic visualization of multi-omic, single-cell sequencing data.

### Implementation

**Fig. 1:**
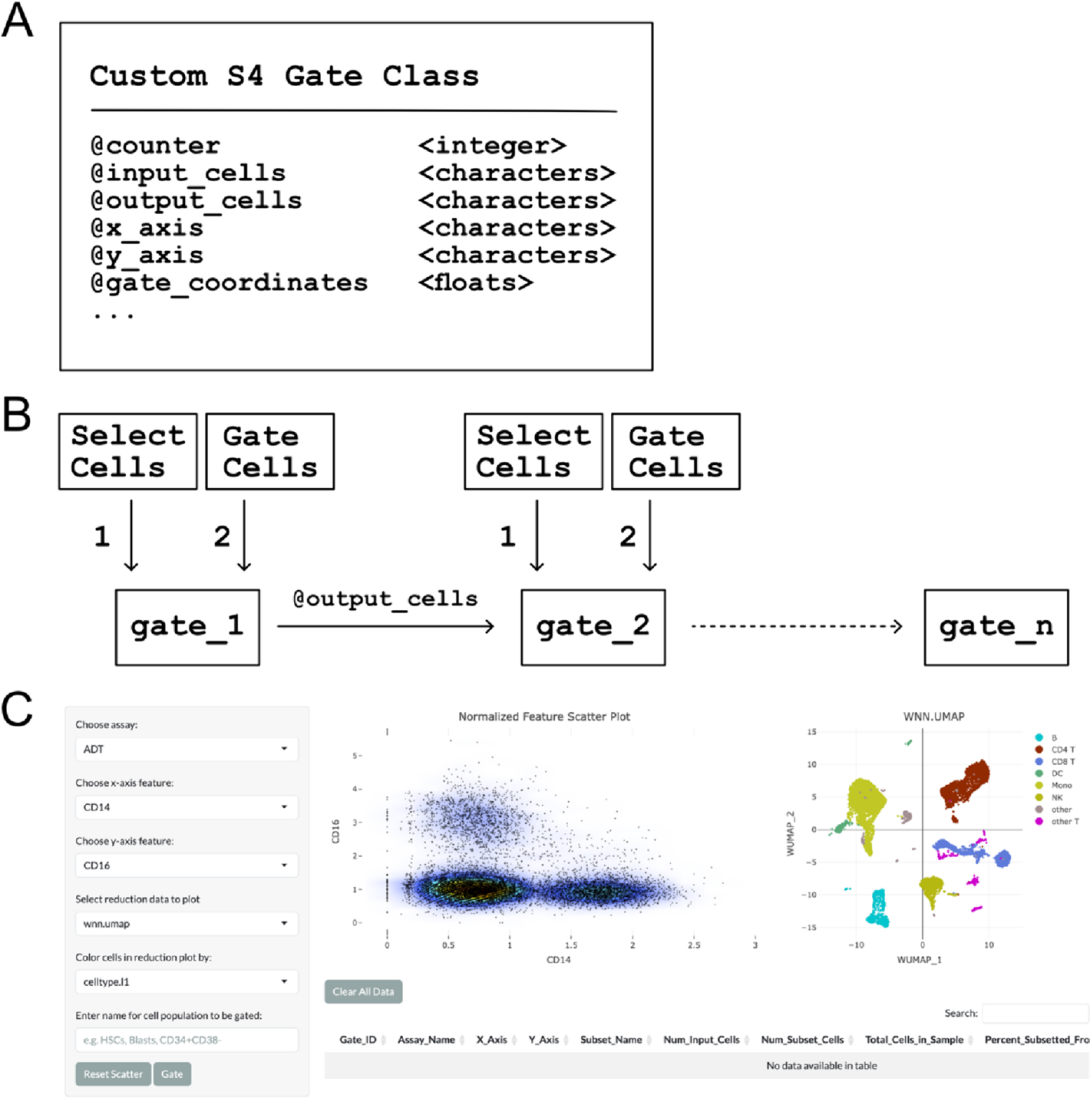
Implementation of CITEViz. **A** Example of a custom Gate class, which contains a counter, input cell barcodes, output cell barcodes, the feature of the x and y axes, gate coordinates, and more. **B** Back-end of CITEViz. Gate classes are created immediately by the input of an interactive cell selection plus the trigger of the Gate button. All attributes of the Gate class are intrinsic to a gate, except for the output cells which are passed between gates. Unlike other packages, this process can be repeated to the nth degree in CITEViz. **C** Screenshot of the gating page in CITEViz with an example PBMC CITE-seq dataset [5]

A core design element of CITEViz is the Gate class—a custom S4 class written in base R (Fig. 1A). In this paper, a “Gate” refers to an R class, while a “gate” refers to a manual selection and filtration of a cell population. A Gate class holds important metadata such as a user-provided gate label, X and Y axes labels (e.g. CD34, CD38), gate selection coordinates, input and output cell barcodes, and more. Most variables are intrinsic to a Gate class except for the output cell barcodes, which are passed between Gates objects to facilitate the gating workflow. Gate classes are created by the simultaneous actions of a Gate button press and a rectangular or polygonal selection of cells in the feature scatter plot (Fig. 1B). The custom Gate class in CITEViz is necessary to store detailed gating information in an organized data structure.

The typical workflow of CITEViz can be distilled down to 4 steps. After uploading a preprocessed Seurat object and choosing the gating tab (Fig. 1C), the user can then

1. Choose which cell surface markers to view in the 2D feature scatter plot on the left pane using a drop-down menu,
2. Select the cells of interest in the scatter plot,
3. Input into the text box a custom label, and
4. Click the “Gate” button to subset the cells of interest

Once a subset of cells is defined, steps 1–4 can be repeated to further analyze the filtered cells. As gates are created, they can be exported as an ordered list of Gate objects, which contains a variety of metadata (e.g. cell barcodes) to facilitate further analyses such as differential expression. In summary, the procedures to gate cells in CITEViz are to plot cell surface markers, select the cells, input a text label, and define the gate via the Gate button.

CITEViz uniquely supports a back-gate function. In the context of CITE-Seq data, back-gates allow users to select cells in latent space and highlight them in the protein feature plots. This feature is useful to explore whether a cell population can be clearly separated by two antibody features, or determine if a better combination of features can properly identify the cell cluster. Cells are initially plotted as gray dots and are colored black based on the selection of cells in the UMAP. The contour plot underneath the dots highlights the density of points. The back-gate function of CITEViz allows users to interactively explore gates and feature combinations that define a specific cell cluster.

At a minimum, CITEViz requires a Seurat object with a normalized ADT counts matrix. Normalization can be performed by the center-log ratio method [6], denoised and scaled by background [7], or a custom method that returns a data matrix under the ADT assay. Since the gating workflow relies on surface protein markers, RNA data normalization is optional but recommended for potential downstream analyses and basic QC visualization.

The two types of input data currently supported by CITEViz are (1) an R Data Serialized (RDS) file containing a Seurat object, or (2) an RDS file with a SingleCellExperiment object derived from the as.SingleCellExperiment() function from the Seurat library. Furthermore, CITEViz can be adapted to accommodate more data structures in future updates (refer to Limitations sub-section in Discussions).

CITEViz provides basic visualization of quality control (QC) metrics, single-feature, and feature co-expression plots. QC metrics include: RNA counts distribution, gene counts distribution, ADT counts distribution, and unique ADT antibodies; these plots can be split by any categorical metadata in the user’s Seurat object. Single feature expression plots allow users to see RNA/ADT expression in latent space, while multi-omic co-expression feature plots can visualize data between assays. This can important to investigate the correlation between transcriptomic expression (RNA) and protein levels (ADT). CITEViz provides basic visualization of QC metrics and single/multi-omic feature expression plots in addition to its primary function as a gating workflow in CITE-Seq data.

## Results

### PBMC CITE-Seq gating analysis

To demonstrate the utility of this program, CITEViz was used to gate the major cell populations in a PBMC CITE-Seq dataset [5]. Several gating schemes were used to show the utility of CITEViz and to identify CD14 monocytes, CD4 T-cells, CD8 T-cells, Natural Killer (NK) cells, B-cells, and platelets. To reduce overplotting, the dataset was randomly down-sampled to 10 K cells and then uploaded to CITEViz.

**Fig. 2:**
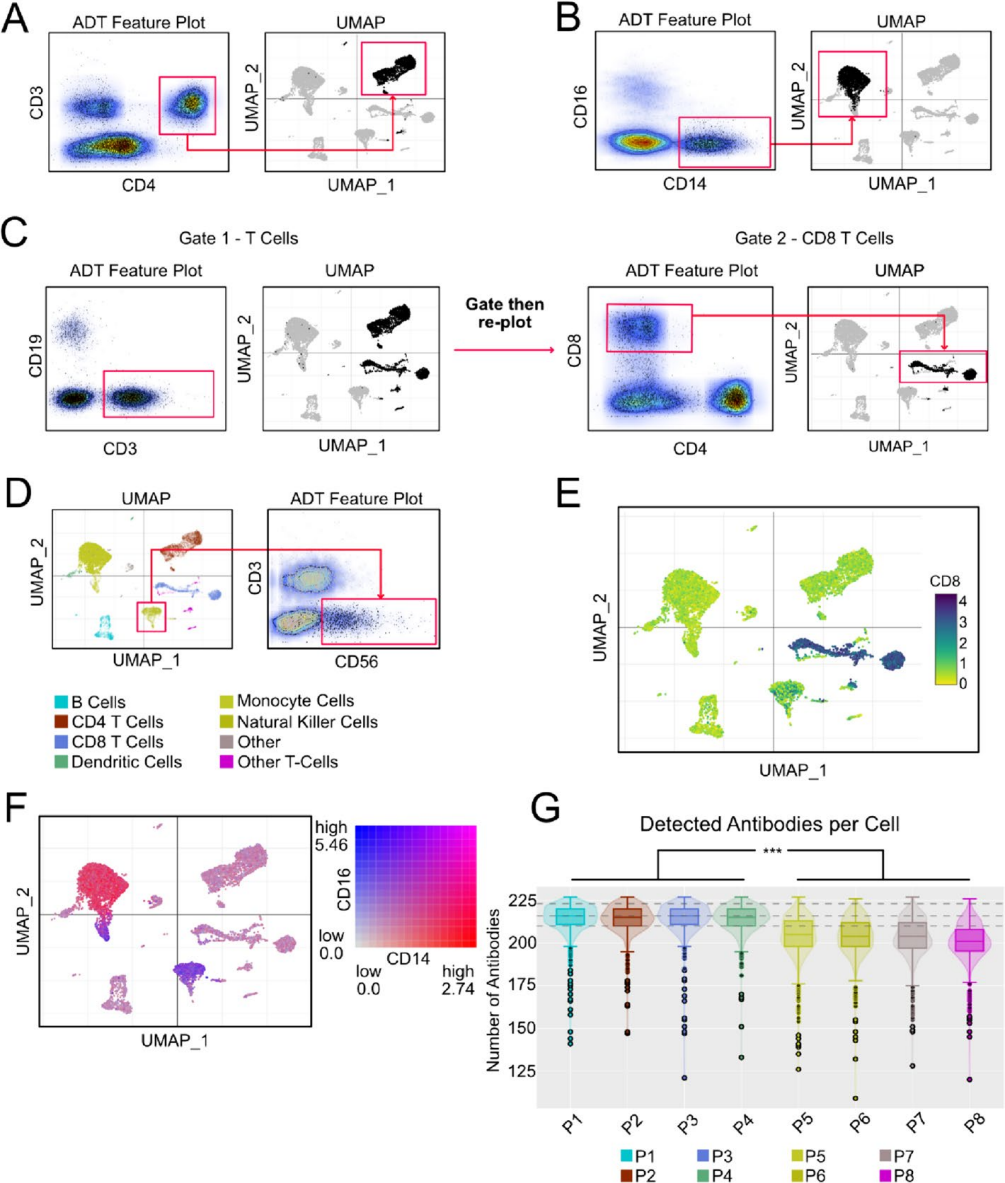
CITEViz analysis of PBMC CITE-Seq data. **A** Identification of CD4-expressing T-cells using CD4-positive and CD3-positive cells, **B** Identification of CD14-expressing monocyte cells with CD14-positive and CD11b-positive markers, **C** 2-layer gate that selects for CD8 T-cells. The first gate consists of CD3-positive cells, followed by a CD4-negative and CD8-positive gate. **D** Example back-gate of natural killer cells shown in an ADT Feature Plot with features of CD3and CD56. **E** Single-feature expression plot of CD8 protein levels. **F** 2-feature co-expression plot using CD16 and CD14 to show population heterogeneity in the monocyte cluster. **G** CITE-Seq QC metric ‘Number of Detected Antibodies per Cell’ split by individual donors

One-step gating schemes were used to identify CD4 T-cells and CD14 monocytes using canonical surface protein markers [4]. Cells with a CD4-positive and CD3-positive protein profile were selected in the ADT Feature Plot, revealing a distinct cell population in the UMAP (Fig. 2A). CD14-expressing monocytes were identified as CD14-positive and CD16-negative cells in the feature scatter plot [8]. The selected cells corresponded to a discrete cell population in the upper left quadrant in the UMAP (Fig. 2B).

CD8 T-cells were identified with a 2-step gating scheme. An initial CD19-negative and CD3-positive gate selected for both CD4 and CD8 T cells (Fig. 2C, left). From this gate, the cells were re-plotted and CD8 T cells were selected for a CD8-positive and CD4-negative protein expression profile [4].

Natural killer (NK) cells were identified in the scatter plots via a back-gate. Within the back-gate tab of CITEViz, NK cells were selected in the latent space according to prior annotations [5]. Once cells were selected, the ADT Feature Plot axes were adjusted to plot CD3 in the y-axis and CD56 in the x-axis based on known NK surface proteins [9]. The corresponding points were displayed in the scatter plot as dark-colored cells expressing a CD56-positive and CD3-negative profile. To identify gates for the remaining major cell populations in the dataset, back-gates for B cells and platelets are included in Additional file 1: Fig. S1. B cells and platelets were also validated using prior [5] and external annotations, respectively [10, 11].

The secondary function of CITEViz is to visualize commonly generated plots in multi-omic CITE-Seq data like single/co-expression plots and QC metrics. For example, we used CITEViz to generate a feature plot, revealing a distinctly high expression in the CD8 T-cell population (Fig. 2E). A protein co-expression feature plot of CD14 and CD16 shows monocyte expressing both markers in the lower arm of the monocyte cluster (Fig. 2F). This pattern was also noticeable in the bulk of the NK cells. An example of a QC metric that can be assessed with CITEViz is the number of unique antibodies detected. CITEViz can split this QC metric by individual patient donors, which clearly displayed a significant difference between patients 1–4 and patients 5–8 with a padj of 0 (Tukey HSD) (Fig. 2G, Additional file 2: Table S1). In conclusion, the ability to visualize multi-omic CITE-Seq data allows for rapid data exploration and assessment of Seurat-processed datasets.

## Discussions

The application of CITEViz to a PBMC CITE-Seq dataset [5] led to the identification of 6 major cell populations (CD4 T cells, CD14 monocytes, CD8 T cells, NK cells, B cells, platelets) using a mix of one- or two-step gates and back-gates (Fig. 2A–D, Additional file 1: Fig. S1). Protein expression data were explored using single and co-expression feature plots, where the latter revealed cellular heterogeneity in CD14 and CD16-expressing monocytes (Fig. 2E, F). By plotting the number of detected antibodies per sample**,** CITEViz displayed a significant difference between patients 1–4 and 5–8. These results are in line with prior analyses and suggest that the flow cytometry-like gating workflow in CITEViz provided an alternative approach to easily classify clusters in CITE-Seq data [5].

### CITEViz compared to other programs

The bioinformatics field, to the best of our knowledge, currently lacks an open-source, R-Shiny package that can (1) streamline the flow cytometry gating process and (2) generate basic multi-omic plots in Seurat-processed CITE-Seq data (Table 1). While the Single-Cell Virtual Cytometer by Pont *et. al.* is most functionally similar to CITEViz, it requires a custom tab-separated input file and is limited to exporting the cell names in the last gated population [12]. The Cascading Style Sheet and JavaScript framework in Pont *et. al.* also can introduce friction with common single-cell analysis packages written in R or Python, thus requiring the user to write additional scripts that import cell barcodes for downstream analyses. In contrast, CITEViz enhances reproducibility by including important gating metadata such as surface protein markers, gate coordinates, and previous filtration steps.

**Table 1:** CITEViz compared to Single-Cell Virtual Cytometer, iSEE, and Seurat

Programs	Gating	Back-Gating	Multi-Omic Feature/QC Visualization	Platform
CITEViz	✓	✓	✓	R-Shiny
iSEE [14]	✓	✓	✓	R-Shiny
Seurat [2]	✓	–	✓	R & R-Shiny
Single-Cell Virtual Cytometer [13]	✓	–	–	Cascading Style Sheet & JavaScript

Another similar tool is the Interactive SummarizedExperiment Explorer (iSEE). iSEE is an R-Shiny package from BioConductor that provides a visual interface to explore single-cell dataset and apply the flow cytometry workflow with minor adjustment of the layout [14]. iSEE was originally optimized for CyTOF and scRNA-Seq data in SingleCellExperiment format but is incompatible with the multi-assay structure of CITE-Seq datasets. To aid users in choosing a particular tool, the main advantage of CITEViz is to implement the gating workflow in CITE-Seq data in native Seurat format via a simplified layout. Users who are looking for a multi-dimensional, tailored exploration of their data with a customizable layout should consider iSEE.

While the flow cytometry gating workflow can be approximated using Seurat [5], the process can be cumbersome and inefficient. The typical workflow to gate cells in Seurat would be to (1) generate a scatter plot with FeatureScatter() and save it as a variable, (2) apply CellSelector() to the feature plot, (3) draw boundaries around the cells of interest in an interactive window, (4) exit the window and repeat steps 1–4 to further filter down cells of interest [5]. A disadvantage of this process is that the gated cells are not immediately highlighted in latent space, resulting in a loss of contextual information. Furthermore, this method requires extensive attention from the user to write redundant code and keep track of many variables. CITEViz improves upon Seurat by displaying relevant parameters and plots in the R-Shiny interface, providing an efficient user experience with continuous visual feedback.

### Limitations

A limitation of CITEViz pertains to data sparsity where gating cells by gene expression (RNA) results in a high rate of data dropout [14]. For example, plotting RNA scatter plots using two transcription factors like IRF8 and CEBPA lead to plots that are difficult to biologically interpret (Additional file 1: Fig. S2). Fortunately, surface protein data (ADT assays) are not sparse and are a reliable resource to characterize cell populations. We speculate the development of better scRNA sequencing assays (and their multi-omic variants) will better resolve heterogeneous cell populations in the future, so CITEViz was built to gate cells based on any assays (RNA, ADT, SCT) and features to accommodate future improvements in sequencing technology.

In addition to sequencing assays, the ever-shifting landscape of bioinformatic file formats can affect compatibility with CITEViz in the future. Currently, CITEViz accepts Seurat and BioConductor SingleCellExperiment objects. To account for the imminent introduction of new file formats or data structures, CITEViz was modularly built to accept various input file types. This is done by various functions that check the input file types and data structures, then building new sub-functions that retrieve the necessary data. The modular design of CITEViz means it can be adequately maintained to accept new file formats and data structures in the future.

### Intended audience

CITEViz is intended to be used by biologists familiar with flow cytometry and who can either (1) perform basic single-cell analysis or (2) collaborate in a team with a computational scientists. The input data format for CITEViz is a pre-processed Seurat object, which requires a basic level of coding skills in R and the ability to follow public Seurat vignettes. Since the essential feature of CITEViz is its iterative filtering process, it is not intended for any data preprocessing or normalization. In our experience, we found CITEViz to be a uniquely collaborative tool that leverages the strengths of both bench and computational scientists to explore and analyze data together.

## Conclusions

CITEViz is an R-Shiny package that facilitates a seamless gating workflow in Seurat-processed CITE-Seq data. Its secondary function is to view basic quality control metrics and multi-omic co-expression plots for data exploration and assessment. By standardizing the gating process, we provide an alternative method for cell cluster classification that is (1) more intuitive for biologists to use, (2) avoids cumbersome and disorganized alternative workflows, and (3) is biologically grounded in established techniques of flow cytometry. CITEViz was ultimately designed to facilitate novel hypothesis generation, and is available to download on GitHub [15].

**Availability and requirements**

**Project name**: CITEViz

**Project home page**: https://github.com/maxsonBraunLab/CITEViz

**Operating system**: Windows/Linux/MacOS

**Programming language**: R

**Other requirements**: R >  = 4.2.0

**License**: MIT

**Any restrictions to use by non-academics**: license needed

## Supplementary Information

**Additional file 1.** Back-gate schemes for B-cells and platelets, and an example gate using scRNA-Seq data.

**Additional file 2.** Tukey Honestly Significant Difference Test of Detected Antibodies per Patient Donor.

## Data Availability

The original data underlying this article are available in GEO (Gene Expression Omnibus) at https://www.ncbi.nlm.nih.gov/geo/, and can be accessed with GSE164378. The dataset analyzed during the current study is available in the Zenodo repository, https://zenodo.org/records/10839852. The CITEViz documentation website can be accessed here https://maxsonbraunlab.github.io/CITEViz/.

## Abbreviations

CITE-Seq: Cellular Indexing of Transcriptomes and Epitopes by Sequencing
scRNA-Seq: Single-Cell RNA-Sequencing
RNA: Ribonucleic acid
ADT: Antibody-Derived Tag
RDS: R Data Serialized
SCT: Single-Cell Transform
PCA: Principal Component Analysis
tSNE: T-Distriuted Stochastic Neighbor Embedding
UMAP: Uniform Manifold Approximation and Projection
PBMC: Peripheral Blood Mononuclear Cells
