# Supplementary material for: CITEViz: interactively classify cell populations in CITE-Seq via a flow cytometry-like gating workflow using R-Shiny
Source: BMC Bioinformatics. 2024 Apr 2;25:142. doi: 10.1186/s12859-024-05762-1 (PMC10988918; doi:10.1186/s12859-024-05762-1)
Supplement: Supplementary file 1 — Additional file 1. Back-gate schemes for B-cells and platelets, and an example gate using scRNA-Seq data. [file 12859_2024_5762_MOESM1_ESM.docx]

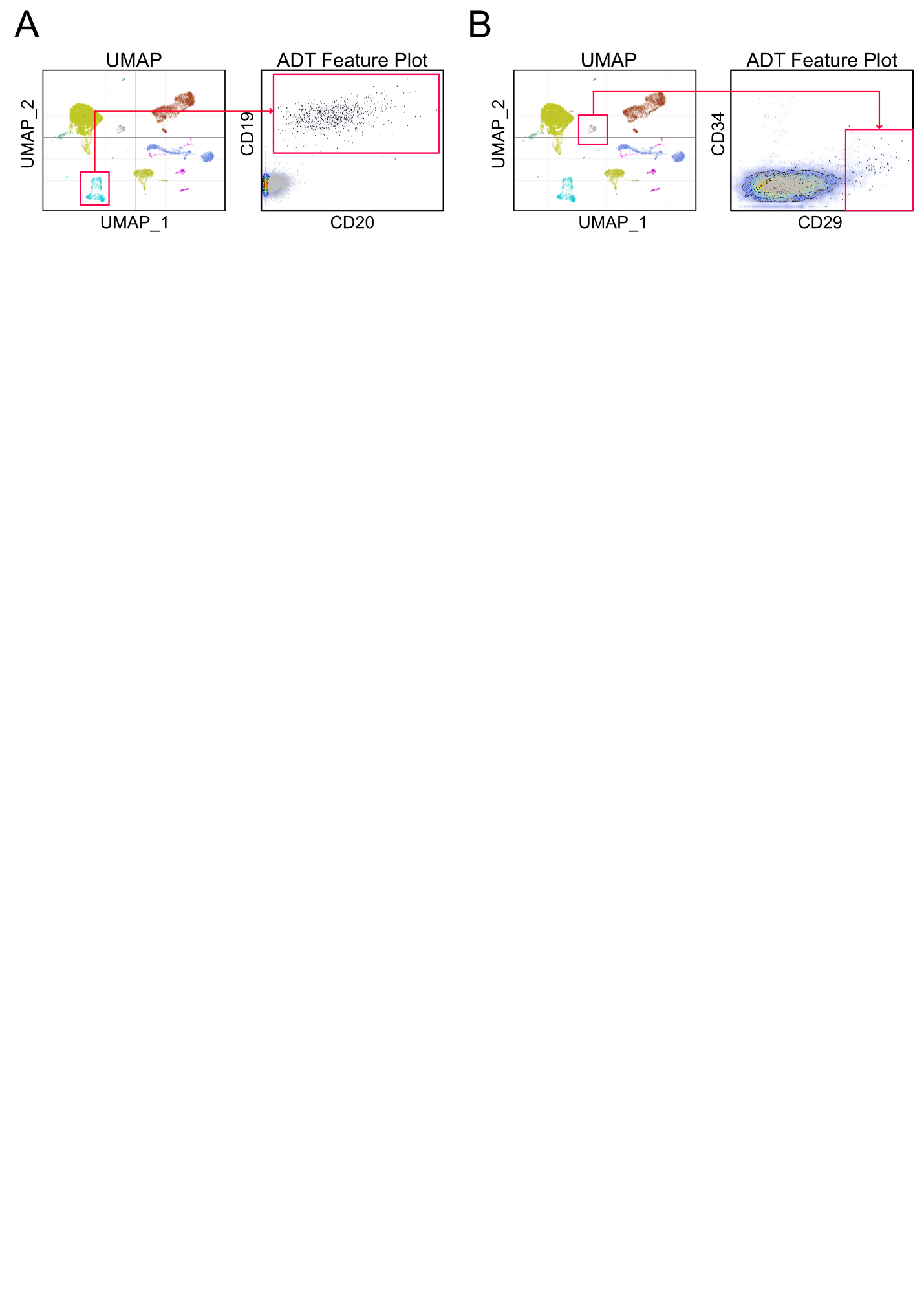


Supplementary Figure 1 – Back-gate of the remaining major cell populations in the PBMC CITE-Seq data. A) B-cells were displayed as CD20-positive and CD19-positive expressing cells. B) Platelet cells were highlighted in the ADT Feature Plot as CD29-positive and CD34-negative.


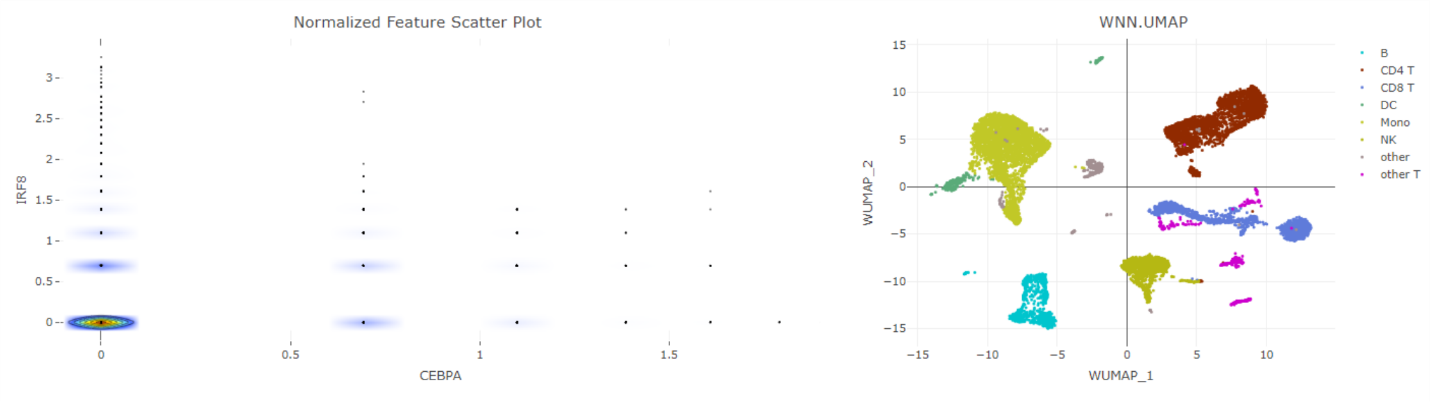


Supplementary Figure 2 – Example of a feature scatter plot to demonstrate sparsity of single-cell transcriptomic data. Gene expression of IRF8 and CEBPA were used to visualize the data. Due to the high dropout rate of single-cell transcriptomic sequencing and low expression of transcription factors, feature scatter plots may not yield an intuitive biological interpretation.
